# Psychosocial and behavioral impact of breast cancer risk assessed by testing for common risk variants: protocol of a prospective study

**DOI:** 10.1186/s12885-017-3485-0

**Published:** 2017-07-18

**Authors:** Tatiane Yanes, Bettina Meiser, Mary-Anne Young, Rajneesh Kaur, Gillian Mitchell, Kristine Barlow-Stewart, Tony Roscioli, Jane Halliday, Paul James

**Affiliations:** 1 Prince of Wales Clinical School, Faculty of Medicine, University of New South Wales, Sydney, NSW 2052 Australia; 2 School of Psychiatry, Faculty of Medicine, University of New South Wales, Sydney, NSW 2052 Australia; 3 Familial Cancer Service, Peter MacCallum Cancer Centre, Melbourne, VIC 3000 Australia; 4 Sir Peter MacCallum Department of Oncology, University of Melbourne, Melbourne, VIC 3052 Australia; 5 Northern Clinical School, Sydney Medical School, University of Sydney, Sydney, NSW 2065 Australia; 6 Department of Medical Genetics, Sydney Children’s Hospital, Sydney, Australia; 7 Public Health Genetics, Murdoch Children’s Research Institute, Melbourne, VIC 3052 Australia; 8 Department of Paediatrics, University of Melbourne, Melbourne, VIC 3052 Australia; 9 Genome.One, Garvan Institute, Sydney, NSW 2010 Australia

**Keywords:** Polygenic risk, Breast cancer, Single nucleotide polymorphism (SNP), Genomic testing, Genetic counselling, Behavioral outcomes, Psychosocial

## Abstract

**Background:**

The ‘common variant, common disease’ model predicts that a significant component of hereditary breast cancer unexplained by pathogenic variants in moderate or high-penetrance genes is due to the cumulative effect of common risk variants in DNA (polygenic risk). Assessing a woman’s breast cancer risk by testing for common risk variants can provide useful information for women who would otherwise receive uninformative results by traditional monogenic testing. Despite increasing support for the utility of common risk variants in hereditary breast cancer, research findings have not yet been integrated into clinical practice. Translational research is therefore critical to ensure results are effectively communicated, and that women do not experience undue adverse psychological outcomes.

**Methods:**

In this prospective study, 400 women with a personal and/or high risk family history of breast cancer will be recruited from six familial cancer centers (FCCs) in Australia. Eligible women will be invited to attend a FCC and receive their personal polygenic risk result for breast cancer. Genetic health professionals participating in the study will receive training on the return of polygenic risk information and a training manual and visual aids will be developed to facilitate patient communication. Participants will complete up to three self-administered questionnaires over a 12-months period to assess the short-and long-term psychological and behavioral outcomes of receiving or not receiving their personal polygenic risk result.

**Discussion:**

This is the world’s first study to assess the psychological and behavioral impact of offering polygenic risk information to women from families at high risk of breast cancer. Findings from this research will provide the basis for the development of a new service model to provide polygenic risk information in familial cancer clinics.

**Trial registration:**

The study was retrospectively registered on 27th April 2017 with the Australian and New Zealand Clinical Trials Group (Registration no: ACTRN12617000594325; clinical trial URL: https://www.anzctr.org.au/Trial/Registration/TrialReview.aspx?id=372743).

**Electronic supplementary material:**

The online version of this article (doi:10.1186/s12885-017-3485-0) contains supplementary material, which is available to authorized users.

## Background

Breast cancer is the greatest cause of premature death in Australian women, accounting for approximately 12% of all premature deaths [1]. Between 10% and 20% of breast cancer is associated with a family history of breast and/or related cancers (termed hereditary breast cancer) [2]. Hereditary breast cancer is clinically important due to the availability of effective risk management strategies that can be targeted to certain subgroups of high-risk women (e.g. breast magnetic resonance imaging and risk-reducing surgery) [3–5].

Since familial cancer clinics (FCCs) were first established in Australia in the early 1990’s, clinical practice has focused on the molecular diagnosis of high-penetrance (*BRCA1/2, TP53, PTEN*) and moderate-penetrance (*PALB2, RAD51C, BRIP1*) pathogenic gene variants, which were discovered through family linkage or candidate gene approaches. However, current testing only identifies a pathogenic gene variant in fewer than 25% of families tested [6], meaning that the majority of families where the risk of hereditary breast cancer is assessed as potentially high receive ‘uninformative’ genetic test results. In these cases the final risk assessment and screening advice is not personalized, but rather based on empiric family history data and extrapolated from population epidemiological studies [7].

The ‘common variant, common disease’ model predicts that a significant component of hereditary breast cancer that cannot be explained by moderate or high-penetrance pathogenic gene variants is due to the cumulative effect of multiple common risk variants in DNA (single nucleotide polymorphisms, SNPs) [8–12]. Individually, each of these common risk variants has only a minimal effect on breast cancer risk, however, when considered altogether, the combined effect is responsible for large differences in risk for different individuals in the population that includes a significantly increased risk for some women. To date more than 96 risk-associated SNPs have been found in large high-quality breast cancer genome-wide association studies [13–16].

The combined effect of common variants is most commonly expressed as a Polygenic Risk Score (PRS). Typically this is calculated by multiplying the risk associated with each SNP that an individual carries, expressed as the per-allele odds ratio, or more commonly adding together the log-odds ratio. Sawyer et al. [9] examined the distribution of the PRS and its clinical implications in the familial breast cancer setting. For this study, breast cancer risk was modeled by genotyping of 22 breast cancer–associated common variants. The study considered a cohort of 954 women with a personal and family history of breast cancer in which a high-risk *BRCA1* or *BRCA2* pathogenic variant had been excluded, and divided them divided into high, intermediate and low polygenic risk groups based on the quartiles of the distribution of the PRS, where the second and third quartiles formed the intermediate risk group. When the features of the three groups were compared, significant differences were identified in the frequency of early-onset and second primary breast cancers. Based on a population lifetime risk of breast cancer of 1 in 11 (9%), the difference in relative risk between low PRS and high PRS was a greater than 4.5-fold, which is equivalent to an average absolute life time breast cancer risk of 6% in the low PRS group and 27% in the high PRS group. Additionally, compared to women with a low PRS, women in the high PRS group had an increased frequency of early onset breast cancers before age 35 years, an approximate two-fold increase in the rate of a contralateral breast cancer, less than half the risk of a *BRCA1/2* mutation, and no increased risk of ovarian cancer [9]. Similar findings have since been reported in additional studies that have incorporated a larger number of common risk variants and combination with risk prediction models [8, 10–12]. In all instances, PRS results have been found to provide a more accurate risk prediction of breast cancer risk than by family history alone.

Current Australian eviQ and UK NICE guidelines recommend enhanced surveillance and risk management strategies for women with a lifetime risk of developing breast cancer over 17% [17, 18]. Thus, women identified as having a high PRS would be eligible for additional risk management strategies, including regular breast screening from a younger age and risk-reducing medication. Additionally, women with a personal history of breast cancer and a high PRS should also be advised about increased risk for contralateral breast cancer and appropriate risk management strategies, including risk-reducing medication if not otherwise indicated by their primary breast cancer pathology, and mastectomy in place of breast conservation. Women who are assessed as intermediate risk by PRS can be advised that their result does not significantly alter their breast cancer risk status, and hence risk management advice is not altered. In contrast, unaffected women assessed as low risk by PRS, can be reassured that population screening levels are appropriate. Where a diagnosis of breast cancer does occur in this group, the lower risk of a second primary cancer may help some women to have confidence to opt for breast conservation. It is important to note however, that for women with a personal diagnosis of breast cancer, a low PRS result does not exclude the possibility of another genetic contribution to their personal history of cancer.

### Psychosocial and Behavioral Outcomes:

The majority of published studies assessing the psychosocial impact of genetic testing for cancer susceptibility have focused on families with a known pathogenic variant in the *BRCA1/2* genes. These studies reported that the uptake of *BRCA1/2* genetic testing is more consistently related to psychological factors (i.e. cancer anxiety and perceived risk) than to sociodemographic variables [19]. Studies on the psychological impact of *BRCA1/2* genetic testing among women demonstrate that non-carriers derive significant psychological benefits from genetic testing and experience few adverse psychological effects, while for carries, distress increases shortly after receiving results but returns to pre-testing levels over time [19–22]. However, one study reported strong declines in well-being in affected women after receipt of testing results [23], indicating that the impact of testing in people affected by cancer is amplified by their experience of cancer.

Regarding its impact on health behaviors, one review article concluded that genetic testing for breast cancer susceptibility is associated with increased adherence to recommended screening and uptake of risk-reducing surgery in affected carriers [24]. In contrast, for those where genetic testing leads to an uninformative test result, studies have reported low uptake of medical and surgical intervention [24]. Further studies in this population have identified that a minority of affected women misinterpret their negative result as meaning that the cancers in their family were definitely not caused by a gene mutation, and hence may feel falsely reassured by their results as ‘No news is good news’ [25]. Thus, testing for common risk variants has the potential to provide personalized risk management recommendations for a significant proportion of at-risk women who would otherwise receive an uninformative result.

To date there has been little research on the uptake and effective communication of this complex polygenic information in the hereditary cancer setting. Early research has been primarily based on hypothetical scenarios assessing interest and attitudes towards testing for common risk variants. These studies have reported a strong interest in polygenic risk testing with interest ranging from 74% to 78% [26–30]. Similarly to uptake of *BRCA1*/2 testing, interest was more consistently related to psychological factors (i.e. perceived risk and greater cancer worry), rather than sociodemographic variables [26–30]. Only two studies have assessed actual uptake of testing and associated outcomes [31, 32]. These studies offered testing for common risk variants associated with colorectal cancer risk; however, they were limited by the small number of variants tested and hence the associated cancer risk was uncertain. The authors concluded that the behavioral changes observed (improvement in diet and exercise) were a result of the genetic counselling, which emphasized lifestyle factors associated with colorectal cancer risk, rather than a result of the polygenic risk information.

Despite increasing support for the utility of common risk variants in hereditary breast cancer [8–12], research has not yet been integrated into clinical practice. Testing for polygenic risk in breast cancer is not currently available in any clinical setting, or currently considered for return to patients outside of a research setting by any FCCs in Australia or internationally. This reflects the status of polygenic risk as an emerging technology and the limited amount of information available on the outcomes of offering such testing. Translational research is needed to develop a model of genetic counselling for polygenic breast cancer risk, which addresses the psychosocial needs of patients and assists health professionals in communicating these complex results to patients.

### Common genomic variants and familial cancer cohort

The Common Genomic Variants and Familial Cancer Study (commonly known as: the Variants in Practice study, ViP) provides a unique cohort in which to systematically ascertain the important psychosocial and clinical implications of testing for polygenic risk and answer a large number of research questions at a small cost [9]. The cohort consists of over 4400 men and women from Victoria and Tasmania, Australia, who have a high-risk family history of breast cancer. Prior to enrolment in the study, all index cases will have attended a participating FCC and undergone clinical assessment, including molecular testing of *BRCA1/2* and other genes depending on their family history and phenotype. Unlike index cases, only a small proportion of family members have a personal history of cancer and most have not attended a FCC. To date 3700 of the total study cohort have had genomic testing for 96 SNPs already known to be associated with breast cancer risk.

### Clinical challenge

The information arising from polygenic risk factors is fundamentally different in nature to testing for monogenic high-penetrance genes, which has traditionally formed the basis of the information provided in FCCs. For example, the interpretation of polygenic risk requires greater consideration of the context, including the individual’s personal and family history, and whether testing for monogenic high-penetrance genes has occurred. In addition, the nature of polygenic inheritance means that breast cancer risk will be present for some women in the absence of a familial pattern. Translational research is critical to ensure that results are effectively communicated, in a way that allows improved risk management strategies to be implemented without undue adverse psychological outcomes. This translational study aims to develop a best-practice model of providing polygenic risk results in the hereditary breast cancer setting, to meet the likely future demand for, and prepare for widespread implementation of genomic testing in this setting.

## Methods/design

### Study objectives and hypotheses

The study will invite 400 female participants from the ViP study (including a mixture of index cases and family members) to receive their personal PRS results and will examine the following aims and hypotheses:

**Aim 1.** To determine the interest in polygenic risk assessment and investigate the determinants of accepting this invitation to receive results, i.e. uptake of this offer and factors associated with uptake.

Hypothesis 1a) Compared to women who decline their results (‘decliners’), women who receive their results (‘receivers’) will:

i. have higher baseline breast cancer anxiety (primary outcome variable), a need to avoid uncertainty, and they will be more likely to have daughters;
ii. be more likely to comply with breast cancer screening guidelines 12 months after receiving their results.

**Aim 2.** Assess the short-(2 weeks) and long-term (12 months) psychological and behavioral outcomes, including compliance with recommended screening and preventative strategies, of ‘receivers’ and ‘decliners’.

Hypothesis 2a) Receivers with a high PRS result will:

i. have increased breast cancer anxiety compared to baseline in the short-term (2 weeks after receiving results), but breast cancer anxiety will return to baseline levels in the long-term (12 months after receiving results); and.
ii. be more likely to report having implemented risk-reducing strategies 12 months after receiving their results when compared to receivers with a low PRS.

Hypothesis 2b) Unaffected women receiving a low PRS will have decreased breast cancer anxiety 2 weeks after receiving results, which will be sustained at 12 months, compared to affected women who receive a low PRS.

Hypothesis 2c) Affected women who receive a high PRS result will exhibit larger increases in breast cancer anxiety from baseline in the short-term (2 weeks after receiving results), compared to unaffected women who receive a high PRS.

### Theoretical framework guiding research

**Fig. 1:**
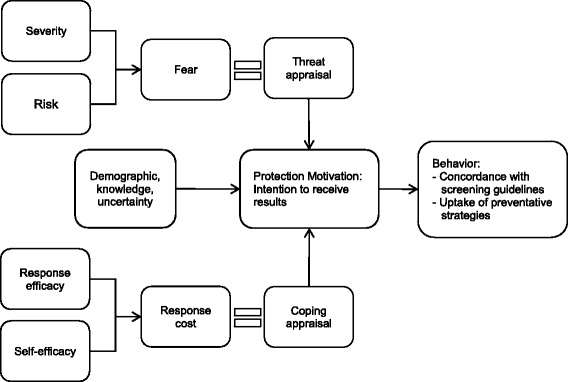
Protection Motivation Framework

Protection Motivation Theory is the theoretical framework guiding this research. This theory has been used to identify the predictors of a range of health behaviors, including uptake of whole genome screening [33, 34]. The theory was developed to address the cognitive processes of individuals that mediate the effect of persuasive communications on behavioral change, through the identification of two independent appraisal processes: threat and coping appraisals. The theory proposes that threat appraisals are based on the individual’s perception of their vulnerability towards, and severity of the undesirable health outcome. Their coping appraisal is centered on the perceived costs of their adaptive response: response efficacy and their own self-efficacy towards partaking in the behavior (Fig. 1).

### Study design

Assessing a woman’s breast cancer risk by profiling common risk variants represents a novel approach in clinical genetics. The PRS results referred to in the protocol are research results obtained from the ViP study and will only be available to the 400 women invited to participate in this psychosocial study.

This is a prospective study which is being conducted across FCCs in two Australian states (Victoria and Tasmania). The study has been approved by the Peter MacCallum Cancer Centre Ethics Committee (HREC/16/PMCC/2) and the Tasmanian Health and Medical Human Research Ethics Committee (H0016395).

The primary psychological outcome measurement is breast cancer anxiety as assessed by the Impact Event Scale (IES). The secondary psychological and behavioral outcomes are: i) general anxiety and depression, ii) test-related distress, positive experiences and uncertainty, iii) concordance with screening guidelines, iv) uptake of preventative strategies, and v) level of decisional regret. The method of determining the PRS has been described elsewhere [9].

**Fig. 2:**
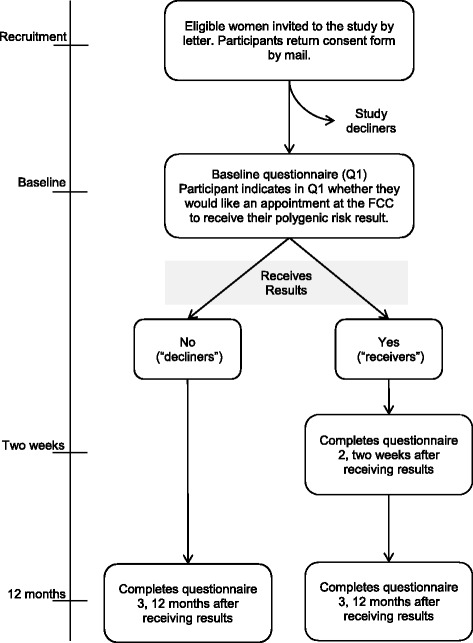
Study design and flow of participants through study

Data will be collected through self-reported questionnaires. Over the course of the study, participants will complete up to three questionnaires. Women who choose to receive their PRS result will complete three questionnaires: at baseline (prior to attending the FCC), two weeks after receiving their PRS result, and 12 months after receiving their result. Women who choose not to receive their result will complete two questionnaires: at baseline and 12 months after enrolment in the study (Fig. 2).

### Participants

#### Inclusion criteria

**Fig. 3:**
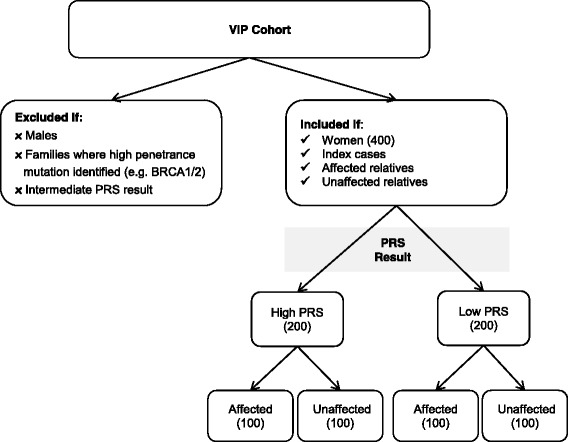
Study inclusion and exclusion criteria

Approximately 400 women will be recruited to this study from the existing ViP cohort. Only women aged 18 years will be recruited. Both index cases and their affected and unaffected family members will be invited to participate in this study. Women will be eligible if they have either a low (*N* = 200) or a high PRS (*N* = 200). Each group will be stratified by disease status, such that about 100 affected and 100 unaffected women are included in each study group (Fig. 3).

#### Exclusion criteria

Women where a pathogenic variant in a moderate or high risk gene has been identified as the cause of cancers in the family will be excluded from the study, as will men, who constitute a very small proportion of index cases (<5%) and relatives (<10%). Men will be excluded from the study as the small sample size will preclude a meaningful statistical comparison with the majority female cohort. Women who receive an intermediate PRS will also be ineligible, because intermediate PRS results do not alter a woman’s risk status and hence risk management advice in a clinically meaningful way. Patients with obvious intellectual or mental impairment that may interfere with the patient’s ability to understand the requirements of the study will also be excluded. Women who are not sufficiently proficient in English to be able to provide written informed consent and complete questionnaires in English will not be recruited to the study.

#### Recruitment

Women selected for inclusion will be invited participate in the psychosocial study by letter. The invitation package will also include a participant information and consent sheet, a response form and a two-page educational pamphlet on genomic testing and breast cancer risk. The educational pamphlet has already been developed and has been pilot-tested with ViP participants to facilitate an informed decision about whether to attend an FCC to receive one’s polygenic risk result (unpublished data).

#### Measures

Women will complete the three self-administered questionnaires over a 12-month period (see Additional file 1). A summary of the measures included at each time point is shown in Table 1.

**Table 1:** Measures selected for study and corresponding questionnaires

Measure	Q1 Baseline	Q2 Receivers	Q3 Receivers	Q3 Decliners
Predictor Variables				
1. Demographic characteristics	√			
2. Protection motivation	√			
3. Perceived severity of breast cancer	√			
4. Response efficacy	√			
5. Response cost	√			
6. Self-efficacy	√			
7. Uncertainty avoidance	√			
Confounding Variable				
8. Stressful life events	√		√	√
Predictor and Outcome Variables				
9. Perceived breast cancer risk	√	√	√	
10. Knowledge of familial breast cancer and polygenic risk	√	√	√	
11. Breast cancer anxiety	√	√	√	√
Outcome Variables				
12. General anxiety and depression	√	√	√	√
13. Concordance with screening guidelines	√		√	√
14. Intention to take up and actual uptake of preventative strategies	√	√	√	√
15. Regret over testing decision		√	√	√
16. Recall and interpretation of results		√	√	
17. Test-related distress, positive experiences and uncertainty		√	√	
18. Reasons for declining results				√

Clinical data available through the ViP study includes: number of affected first- and second-degree relatives, including number deceased due to breast cancer, personal history of breast cancer, and for affected women, time since diagnosis.

#### Predictor variables

1. *Demographic characteristics –* sociodemographic data to be collected includes age, gender, country of origin, marital status, educational level, income, language spoken at home, number of biological children, and previous attendance at an FCC.
2. *Protection motivation –* one 7-point Likert-type item will assess intention to receive PRS result.
3. *Perceived severity of breast cancer* – will be assessed with one item adapted from a previous study [35].
4. *Response efficacy –* six items were adapted from [35] to assess perceived benefits of receiving one’s PRS. Participants will be asked to rate from ‘not at all’ (1) to ‘very much’ (3) the extent to which different factors have influenced their decision to access their PRS result (e.g. learn about my children’s risk, to plan for the future).
5. *Response cost –* six items were adapted from [35] to assess perceived disadvantages to receiving a PRS result. Participants will be asked to rate from ‘not at all’ (1) to ‘very much’ (3) the extent to which different factors have influenced their decision not to access their result (e.g. concern about the impact of genetic information on my family, possible impact on insurance).
6. *Self-efficacy –* will be measured with seven items to assess confidence in undertaking SNP testing despite ‘obstacles’. Participants will be asked to rate their agreement from ‘strongly disagree’ (1) to ‘strongly agree’ (5) with statements such as ‘I am confident I can receive my genomic testing result even if’…‘my family did not want me to, I had to communicate the results to my family’ [34].
7. *Uncertainty avoidance* – will be assessed using the eight-item Attitudes Towards Uncertainty scale [36], which has previously demonstrated high internal reliability [34]. The eight items are measured on a five-point scale ranging from ‘strongly disagree’ (1) to ‘strongly agree’ (5), with higher scores indicating a more negative attitude towards uncertainty.

#### Confounding variable

8. *Stressful life events*: will be assessed using the 12-item List of Threatening Experiences, which measures common threatening life experiences, including serious illness and death in the family [37]. Threatening life events may affect anxiety and distress levels and will be measured as potential confounding variable.

#### Predictor and outcome variables

9. *Perceived breast cancer risk* – will be measured with three items used in a previous study [35].
10. *Knowledge of familial breast cancer and polygenic risk* – 10 true-false items have been developed to assess knowledge of polygenic inheritance and hereditary breast cancer.
11. *Breast cancer anxiety:* will be measured using the Impact of Events Scale (IES), a measure of intrusion and avoidance toward a stressor, in this case being at risk for breast cancer [38]. The IES consists of 15 items with response options ranging from ‘not at all’ (0) to ‘often’ (5). A total score is obtained by summing the items (range 0 to 75) with a higher score indicating more distress [38]. The IES has been validated in similar populations [39].

#### Outcome variables

12. *General anxiety and depression:* will be assessed using the Hospital Anxiety and Depression Scale (HADS). The 14-item HADS is a widely used measure of emotional disturbance and has two subscales measuring general anxiety and depression [40]. Each question has four possible responses, with responses scored on a scale from 0 to 3. A total scale score is obtained by summing each item (range 0 to 42) with a higher score indicating more general anxiety and depression.
13. *Concordance with screening guidelines* – six items have been developed in concordance with national guidelines for mammography and clinical breast examination [41] screening using the approach used in a previous study [42]. Participants will be categorized in terms of their concordance to the current screening guidelines.
14. *Intention to take up and actual uptake of preventative strategies –* 15 items have been developed to assess intention and uptake of preventative strategies, including risk-reducing surgery (bilateral mastectomy), medication (i.e. tamoxifen and raloxifen), and lifestyle factors (i.e. alcohol consumption, and exercise).
15. *Regret over testing decision* – will be assessed using the five-item Decision Regret Scale, which correlates with decisional conflict and quality of life [43].
16. *Recall and interpretation of testing results* – three items have been developed to assess recall and understanding of testing results.
17. *Test-related distress, positive experiences and uncertainty –* this measure includes 19 items from a validated questionnaire, the Multidimensional Impact of Risk Assessment Scale [44], assessing distress (six items), positive experiences (four items), and uncertainty (nine items) about genetic testing. Response options range from ‘never’ (0) to ‘often’ (5) with higher scores indicating higher psychological distress.
18. *Reasons for declining results* – will be assessed with 15 items used in a previous study [18]. Women will be asked to indicate the extent to which possible reasons for declining to receive results apply to them.

#### Genetic counseling consultation and disclosure of results

In line with clinical care practice, participants will receive their PRS result by attending an in-person appointment with a qualified genetic health professional (genetic counselor and/or medical geneticists) at one of the participating FCCs. As the return of polygenic information represents a novel practice in genetic counselling, genetic health professionals at each of the participating FCC will receive training on polygenic inheritance. A training manual will also be developed covering: interpretation of PRS results and current research, genetic counseling frameworks for polygenic inheritance [27, 31, 45, 46], impact on risk management options, implications for family members, and potential psychosocial implications.

To measure consultation characteristics, a brief consultation report will be completed after each appointment which includes: participant’s PRS result, type of cancer (for affected women), recommended risk management strategies, number of occasions of service, length of consultation, and health professionals involved in the consultation.

#### Sample size and power

Based on similar previous studies [35, 47], a sample size of 400 women is required to have 320 women completing the study with 215 receivers and 105 decliners (after adjusting for loss to follow up of approximately 20%). For a two sided test and based on a 5% significance level, this sample size will have 80% power to detect a clinically meaningful difference in the primary psychological outcome of breast cancer anxiety as measured by the IES (SD 14.2, range 0–75 scores) [48] at the 2-week follow-up, between affected and unaffected women who receive a high PRS result (hypothesis 2c). A difference of seven scores (half a standard deviation) on the IES is considered a clinically meaningful difference to detect [49].

#### Statistical analyses

For each of the main outcome variables (e.g. breast cancer anxiety), linear or logistic regression will be used as appropriate. Further multivariable analyses will be used to adjust for potential confounding variables (e.g. age, parity, stressful life events). Appropriate regressions will be performed to investigate whether outcomes differ between receivers and decliners (hypothesis 1a) and between subgroups of affected and unaffected women (hypothesis 2c) and those receiving either a low or high PRS (hypothesis 2b and 2c). Repeated measurements will be analyzed using linear mixed models to assess how outcomes change over time among receivers (hypothesis 2ai and 2b). This approach adjusts for the repeated measures per person and also allows for missing values.

## Discussion

To our knowledge this is the world’s first study to assess determinants for uptake of polygenic risk information, and the psychological and behavioral impact of receiving this information. Testing for polygenic risk will result in a paradigm shift in the practice of clinical genetics and oncology. Currently, genetic testing for hereditary cancer is offered in relation to personal and family cancer history, cancer type and/or other clinical criteria based on the likelihood of a pathogenic variant in high- penetrance gene being present, and these genes form the sole basis of the test. Because of this, the majority of women attending FCCs for hereditary breast cancer are not offered testing as they do not meet the minimum criteria for genetic testing. However, it is increasingly clear that breast cancer risk is also associated with other types of genetic risk (e.g. polygenic risk), often in the absence of additional family history for those women who are already affected by cancer. The inclusion of polygenic risk in FCCs will dramatically change service provision and allow access to personalized genetic testing to a wider group of women, including testing of women with breast cancer unselected for family history.

Findings from this study will also have implications for testing for common risk variants in other settings (e.g. hereditary cardiovascular disease and diabetes) and this study will provide a model for similar research across other important fields in medicine which are impacted by genomics.

### Methodological strengths and limitations

A substantive strength of this study is the large and diverse cohort available through the parent ViP study. The parent study aims to recruit every family in Victoria and Tasmania that attended a FCC to undergo genetic testing for hereditary breast cancer. The multicenter approach and diverse cohort will increase the external validity and generalization of the study findings. The sample size in the current study will provide sufficient power to detect clinically meaningful effects for the key outcome variable of breast cancer anxiety.

The study is a prospective study, which employs, wherever possible, validated measures that have been utilized previously with women at high risk for breast cancer. In applying this study design we hope to build a comprehensive picture of the psychological and behavioral outcomes associated with receiving polygenic breast cancer risk information.

Two potential limitations of the study must also be acknowledged. Firstly, it was beyond the capacity of the research to translate the patient questionnaires into other languages. Hence, women from non-English speaking backgrounds cannot be included. Secondly, this study will not involve development and assessment of pre-testing genetic counselling as the PRS results are available as part of the parent ViP study. The focus of this translational research is to explore the uptake of PRS results and psychological and behavioral outcomes associated with receiving or not receiving one’s PRS result. Future research will be able to explore pre-testing genetic counselling and the informed consent process including the provision of information of the benefits and limitations of SNP testing.

## Additional file

Additional file 1: Study Questionnaires. This file contains all the study's questionnaires including baseline, short term, and long term questionnaire for receivers and decliners. (PDF 1051 kb)

## Abbreviations

BRCA1/2: Breast cancer gene 1 and 2
FCC: Familial cancer clinic
PRS: Polygenic risk score
SNPs: Single nucleotide polymorphism
