# Supplementary material for: Psychosocial and behavioral impact of breast cancer risk assessed by testing for common risk variants: protocol of a prospective study
Source: BMC Cancer. 2017 Jul 18;17:491. doi: 10.1186/s12885-017-3485-0 (PMC5516374; doi:10.1186/s12885-017-3485-0)
Supplement: Additional file 1: — Study Questionnaires. This file contains all the study's questionnaires including baseline, short term, and long term questionnaire for receivers and decliners. (PDF 1051 kb) [file 12885_2017_3485_MOESM1_ESM.pdf]

Questionnaire One

We are interested in finding out about your thoughts and the factors associated, with choosing to know or not know your genomic testing results for common risk variants associated with breast cancer risk. To help us with this, we ask that you complete the following questionnaire.

About the questionnaire

All the information you provide will be treated as *strictly confidential* and your identity will never be revealed in any reports. The completed questionnaires will be kept separately from any information that could identify you and will be kept securely under lock and key. There is no need for you to write your name on this questionnaire. There are no right or wrong answers, and we ask you simply to tick those answers that most apply to you.

<u>Participation in this study is entirely voluntary</u>; you are not obliged to participate and if you do participate you can withdraw at any time. Whatever your decision, it will not affect your relationship with your treating doctors or hospital in case you are currently being treated for breast cancer or your relationship with the researchers involved in the 'Common Genetic Variants and Familial Breast Cancer Study'.

You may feel that some of the questions we ask are stressful or upsetting. If you become upset or you do not wish to answer a question, you may skip it and go to the next question, or you may stop immediately. If you become upset or distressed as a result of your participation in this study, the research team will arrange for counselling or other appropriate support. Any counselling or support will be provided by qualified staff members who are not members of the research team. This counselling will be provided free of charge

When you have completed the questionnaire, please post the questionnaire to us by using the enclosed reply paid envelope within the next ten days, if possible.

Would you like to receive a free summary report of the findings?

❑1 No ❑2 Yes

Thank you very much for your help in this important study

Registration Number: ________________

Date Issued: ________________

Section 1: Background questions

The first section of the questionnaire asks some general background questions which will be helpful to us in analysing the data. It will not be used for identification.

1. What is your age: _________ (years)

2. What is your country of origin?
   - ❑ 1 Australia
   - ❑ 2 Other, please specify:

   ____

3. When you are at home, what language do you mostly speak?
   - ❑ 1 English
   - ❑ 2 Other, please specify:

   ____

4. What is your current marital status?
   - ❑ 1 Never married
   - ❑ 2 Married or de facto
   - ❑ 3 Widowed
   - ❑ 4 Separated but not divorced
   - ❑ 5 Divorced
   - ❑ 6 Other, please specify::

   ____

5. What is the highest level of education you have completed?
   - ❑ 1 None
   - ❑ 2 Primary School
   - ❑ 3 High School
   - ❑ 4 TAFE or College certificate or diploma
   - ❑ 5 Bachelor's degree
   - ❑ 6 Postgraduate qualification
   - ❑ 7 Other, please specify:

   ____

6. Do you have **biological** children?
   - ❑ 1 No (go to question 8)
   - ❑ 2 Yes

7. How many daughters do you have?

   ____

8. What is your occupation (if retired, past occupation)?

   ____

9. What is your current employment status? (Please tick the box that best describes your employment status)
   - ❑ 1 Full-time employed
   - ❑ 2 Part-time employed
   - ❑ 3 Unemployed
   - ❑ 4 Self-employed
   - ❑ 5 Homemaker
   - ❑ 6 Full-time student
   - ❑ 7 Part-time student
   - ❑ 8 Permanently unable to work
   - ❑ 9 Retired
   - ❑ 10 Other, please specify:

   ____

10. Have you ever attended a family cancer clinic to discuss your personal and/or family history of cancer?
    - ❑ 1 No
    - ❑ 2 Yes
    - ❑ 3 Unsure

11. How much have you heard or read about genomic testing for common risk variants?
    - ❑ 1 Nothing
    - ❑ 2 Almost nothing
    - ❑ 3 Some information
    - ❑ 4 A fair amount of information
    - ❑ 5 A lot of information

Section 2: Chances of Developing Breast Cancer

In this section, we are interested to know **your opinion** about breast cancer, and your chances of developing breast cancer in the future.

12. Please rate how much you agree with the following statement:

    How serious would it be if you were diagnosed with breast cancer someday (or another breast cancer if you have been previously diagnosed with breast cancer)

**Not serious at all**		**Somewhat serious**		**Extremely serious**
1	2	3	4	5

13. What do you think are your chances of developing breast cancer sometime in the future compared to:

    a. an average person the same age and gender as you?

**Much lower**	**Lower**	**Same**	**Higher**	**Much Higher**
1	2	3	4	5

    b. an average person the same age and gender as you **AND** with a similar family history breast cancer as you?

**Much lower**	**Lower**	**Same**	**Higher**	**Much Higher**
1	2	3	4	5

14. Based on your own opinion, please rate your chances of developing breast cancer sometime in the future on a scale of 0–100%, where 0% = no chance of ever developing breast cancer, and 100% = will definitely develop breast cancer someday.

    Please do this by drawing a **mark, like this: |** **anywhere** along the line below:

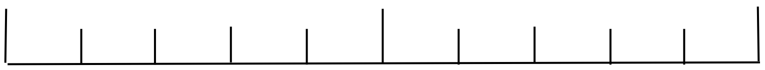

0% — No chance of developing breast cancer

50% — 50-50 (or 1 in 2) chance of developing breast cancer

100% — Will definitely develop breast cancer

Section 3: Knowledge of Breast Cancer Risk

In this section we would like to find out what you know about DNA variations and breast cancer risk.

15. For each of the following statements please indicate if you believe it to be True, False or Don’t Know. If you are unsure or don’t know the answer, please tick the “don’t know” box.

	**False**	**True**	**Don’t Know**
All women at high risk for breast cancer will develop breast cancer	❑1	❑2	❑3
The interpretation of a high or low risk is the same for everyone	❑1	❑2	❑3
If a woman has a high risk for breast there are screening and preventative options available	❑1	❑2	❑3
Common risk variants associated with breast cancer risk also increases a woman’s risk for ovarian cancer	❑1	❑2	❑3
There is more than one DNA change that can increase a woman’s risk for breast cancer	❑1	❑2	❑3
It is possible to be diagnosed with breast cancer solely due to chance	❑1	❑2	❑3
Most women who develop breast cancer do not have family history of the disease	❑1	❑2	❑3
A woman can only inherit DNA changes associated with breast cancer risk from her mother	❑1	❑2	❑3
A woman may be at increased risk for breast cancer if she has several close relatives with breast cancer	❑1	❑2	❑3
If a woman learns that she does not have a fault in the *BRCA1* or *BRCA2* genes, that means the breast cancer in her family cannot be hereditary	❑1	❑2	❑3

Section 4: Thoughts about Genomic Testing

This section looks at your thoughts and feelings about receiving the results of your genomic testing to assess breast cancer risk.

Please indicate the extent to which you agree or disagree with each of the statements below.

16. I am confident I could receive my genomic testing result even if:

	Strongly disagree	Disagree	Neither agree or disagree	Agree	Strongly agree
…my family did not want me to	❑1	❑2	❑3	❑4	❑5
…the results cannot be explained by current scientific knowledge about genes and disease	❑1	❑2	❑3	❑4	❑5
…I had to attend genetic counselling before and after receiving my results	❑1	❑2	❑3	❑4	❑5
…the results could uncover unwanted information about my disease risk	❑1	❑2	❑3	❑4	❑5
…I had to pay for an appointment	❑1	❑2	❑3	❑4	❑5
…I had to communicate the results to my family	❑1	❑2	❑3	❑4	❑5
…I had to communicate the results to insurance providers/future employer	❑1	❑2	❑3	❑4	❑5

Please continue over the page

17. Below is a list of reasons other people have given for their decision **to access** their genomic information.

Please indicate how much each of these reasons would influence, or has influenced, your personal decision to access your genomic testing results.

	**Not applicable**	**Not at all**	**Somewhat**	**Very much**
To learn about my children's cancer risk.	❑1	❑2	❑3	❑4
To get information about how to manage my risk of developing cancer.	❑1	❑2	❑3	❑4
To plan for the future.	❑1	❑2	❑3	❑4
To allow family members to learn about their cancer risk.	❑1	❑2	❑3	❑4
To help research.	❑1	❑2	❑3	❑4
To confirm my belief that I have a genetic change related to cancer.	❑1	❑2	❑3	❑4
To be more certain about my cancer risk.	❑1	❑2	❑3	❑4
To have the information for decisions about having children in the future.	❑1	❑2	❑3	❑4

18. Below is a list of reasons other people have given for their decision **not to** access their genomic information.

Please indicate how much each of these reasons would influence, or has influenced your personal decision **not to** access your genomic testing results.

	**Not applicable**	**Not at all**	**Somewhat**	**Very much**
Concern about the impact of the genomic information on my family members	❑1	❑2	❑3	❑4
It wouldn't change my belief that cancer is inevitable	❑1	❑2	❑3	❑4
Concern about coping with the information emotionally	❑1	❑2	❑3	❑4
Concern that the information is not accurate	❑1	❑2	❑3	❑4
The possible impact on insurance	❑1	❑2	❑3	❑4
The hassle of attending an appointment at the family cancer clinic	❑1	❑2	❑3	❑4
It conflicts with my religious, cultural and/or spiritual beliefs	❑1	❑2	❑3	❑4

Are there any **other** factors that may influence your decisions about receiving or not receiving your genomic testing results? Please specify the factor(s) and how it has influenced your decision.

____

____

____

____

____

____

____

____

____

19. Below is a list of comments made by people when deciding whether to receive their genomic testing results to estimate breast cancer risk.

    Please indicate how much you agree or disagree with each statement by **ticking** the number that best represents **your** views

	**Strongly disagree**	**Disagree**	**Neither agree or disagree**	**Agree**	**Strongly agree**
I would rather receive my genomic testing results, and be certain about my future health, even if the result is bad news	❑1	❑2	❑3	❑4	❑5
I would like to know now if I am likely to develop breast cancer so I can get used to the news	❑1	❑2	❑3	❑4	❑5
If I didn't receive my genomic testing results I would always be wondering whether I was going to develop the breast cancer	❑1	❑2	❑3	❑4	❑5
The relief I would get from a good result makes it worth the risk that the result is bad	❑1	❑2	❑3	❑4	❑5
I think it is tempting fate to ask questions about future illness	❑1	❑2	❑3	❑4	❑5
I would rather live with uncertainty, than find out I was going to develop breast cancer	❑1	❑2	❑3	❑4	❑5
Knowing the result of my genomic testing would mean I felt more in control	❑1	❑2	❑3	❑4	❑5
It is better to know that I will develop breast cancer, even if I can't prevent it	❑1	❑2	❑3	❑4	❑5

Section 5: Thoughts and Feeling

20. Below is a list of comments made by people during various life events.

Please tick the box corresponding to the statement that indicates how frequently each comment was true for you **<u>in the past week regarding your chances of developing breast cancer</u>.** If any of these responses did not occur, tick the “not at all” box.

	**Not at all**	**Rarely**	**Sometimes**	**Often**
I thought about it when I didn't mean to	❑0	❑1	❑3	❑5
I avoided letting myself get upset when I thought about it or was reminded of it	❑0	❑1	❑3	❑5
I tried to remove it from my memory	❑0	❑1	❑3	❑5
I had trouble falling asleep or staying asleep because of pictures or thoughts about it that came into my mind	❑0	❑1	❑3	❑5
I had waves of strong feelings about it	❑0	❑1	❑3	❑5
I had dreams about it	❑0	❑1	❑3	❑5
I stayed away from reminders of it	❑0	❑1	❑3	❑5
I felt as if it hadn’t happened or wasn't real	❑0	❑1	❑3	❑5
I tried not to talk about it	❑0	❑1	❑3	❑5
Pictures about it popped into my mind	❑0	❑1	❑3	❑5
Other things kept making me think about it	❑0	❑1	❑3	❑5
I was aware that I still had a lot of feelings about it, but I didn't deal with them	❑0	❑1	❑3	❑5
I tried not to think about it	❑0	❑1	❑3	❑5
Any reminder brought back feelings about it	❑0	❑1	❑3	❑5
My feelings about it were kind of numb	❑0	❑1	❑3	❑5

This section is designed to help us know how you feel. Please read each item below, and place a tick in the box which comes closest to how you have been feeling in the **PAST WEEK**. Don't take too long over your replies; your immediate reaction to each item will probably be more accurate than a long thought out response.

21. I feel tense or 'wound up':
- ❑ 1 Most of the time
- ❑ 2 A lot of the time
- ❑ 3 From time to time, occasionally
- ❑ 4 Not at all

22. I still enjoy the things I used to enjoy:
- ❑ 1 Definitely as much
- ❑ 2 Not quite so much
- ❑ 3 Only a little
- ❑ 4 Hardly at all

23. I get a sort of frightened feeling as if something awful is about to happen:
- ❑ 1 Very definitively and quite badly
- ❑ 2 Yes, but not too badly
- ❑ 3 A little, but it doesn't worry me
- ❑ 4 Not at all

24. I can laugh and see the funny side of things:
- ❑ 1 As much as I always could
- ❑ 2 Not quite so much
- ❑ 3 Definitely not so much now
- ❑ 4 Not at all

25. Worrying thoughts go through my mind:
- ❑ 1 A great deal of the time
- ❑ 2 A lot of the time
- ❑ 3 From time to time but not too often
- ❑ 4 Only occasionally

26. I feel cheerful:
- ❑ 1 Not at all
- ❑ 2 Not often
- ❑ 3 Sometimes
- ❑ 4 Most of the time

27. I can sit at ease and feel relaxed:
- ❑ 1 Definitely
- ❑ 2 Usually
- ❑ 3 Not often
- ❑ 4 Not at all

28. I feel as if I am slowed down:
- ❑ 1 Nearly all the time
- ❑ 2 Very often
- ❑ 3 Sometimes
- ❑ 4 Not at all

29. I get a sort of frightened feeling like butterflies in my stomach:
- ❑ 1 Not at all
- ❑ 2 Occasionally
- ❑ 3 Quite often
- ❑ 4 Very often

30. I have lost interest in my appearance:
- ❑ 1 Definitely
- ❑ 2 I don't take so much care as I should
- ❑ 3 I may not take quite as much care
- ❑ 4 I take just as much care as ever

31. I feel restless as if I have to be on the move:
- ❑ 1 Very much indeed
- ❑ 2 Quite a lot
- ❑ 3 Not very much
- ❑ 4 Not at all

32. I look forward with enjoyment to things:
- ❑ 1 As much as I ever did
- ❑ 2 Rather less than I used to
- ❑ 3 Definitely less than I used to
- ❑ 4 Hardly at all

33. I get sudden feelings of panic:
- ❑ 1 Very often indeed
- ❑ 2 Quite often
- ❑ 3 Not very often
- ❑ 4 Not at all

34. I can enjoy a good book or radio or TV program:
- ❑ 1 Often
- ❑ 2 Sometimes
- ❑ 3 Not often
- ❑ 4 Very seldom

35. Below is a list various stress life events that that people can experience.

Please tick the box below, either 'Yes' or 'No' to indicate if any of the events below occurred for **<u>you in the past year.</u>**

	No	Yes
You yourself suffered a serious illness (including a new cancer diagnosis), injury or an assault	❑1	❑2
A serious illness (including cancer diagnosis), injury or assault happened to a close relative	❑1	❑2
Your parent, child or spouse died	❑1	❑2
A close family friend or another relative (aunt, cousin, grandparent) died	❑1	❑2
You had a separation due to marital difficulties	❑1	❑2
You broke off a steady relationship	❑1	❑2
You had a serious problem with a close friend, neighbour or relative	❑1	❑2
You became unemployed or you were seeking work unsuccessfully for more than one month	❑1	❑2
You were sacked from your job	❑1	❑2
You had a major financial crisis	❑1	❑2
You had problems with the police and a court appearance	❑1	❑2
You moved to a new house	❑1	❑2
Something you valued was lost or stolen	❑1	❑2

Section 6: Breast Cancer Risk Management

This section looks at your current strategies to manage your breast cancer risk. Not all options may be relevant to you. However, they may be discussed during an appointment with at a family cancer clinic. Please note, that your responses to the following questions will not limit your options in the future.

36. Do you examine your breasts yourself?
    - ❑ 1 No, please go to question 38
    - ❑ 2 Yes

37. If you have answered yes, how often have you examined your breasts over the **past year**?
    - ❑ 1 Less frequently than monthly
    - ❑ 2 Monthly
    - ❑ 3 More frequently than monthly

38. Have you ever had a doctor or nurse examine your breasts?
    - ❑ 1 No, please go to question 40
    - ❑ 2 Yes

39. If you have answered yes, how often have you had a doctor or nurse examine your breasts over the **past year**?
    - ❑ 1 Less frequently than once a year
    - ❑ 2 Once a year
    - ❑ 3 More frequently than once year

40. Have you ever had any screening tests for breast cancer? Please tick any that apply.
    - ❑ 1 Not applicable, I had double mastectomy, please go question 45
    - ❑ 2 No, please go question 42
    - ❑ 3 Yes, I have had screening tests for breast cancer

41. If you have answered yes, please indicate what screening test(s) you have and the year of your most recent test:
    - ❑ 1 Mammogram (an X-ray of the breast for women with no breast symptoms)
      Year: __________
    - ❑ 2 Breast ultrasound
      Year: __________
    - ❑ 3 Breast MRI (or magnetic resonance imaging, a test that uses a magnetic field to view the breast tissue).
      Year: __________

42. Are you planning to change your current screening for breast cancer?

- ❑ 1 No, I already have the recommended screening for my breast cancer risk
- ❑ 2 Yes I planning to have **more** frequent screening tests
- ❑ 3 Yes I planning to have more **less** frequent screening tests

43. Have you had a bilateral mastectomy (surgery to remove both breasts)?

- ❑ 1 No, I have not had this surgery
- ❑ 2 Yes, please go to question 45

44. Are you planning to have a bilateral mastectomy to reduce your breast cancer risk?

- ❑ 1 No, I am not planning to have this surgery
- ❑ 2 Yes, I am planning having this surgery in the next 12 months
- ❑ 3 Yes, I am planning to have this surgery but **not** in the next 12 months

45. Do you currently take medication to reduce your breast cancer risk (e.g. Tamoxifen)?

- ❑ 1 No, I do not take medication
- ❑ 2 Yes, please go to question 47

46. Are you planning to take medication to reduce your breast cancer risk?

- ❑ 1 No, I am not planning to take risk reducing medication
- ❑ 2 Yes, I am planning to take risk reducing medication in the next 12 months
- ❑ 3 Yes, I am planning to take risk reducing medication **but not** in the next 12 months

The questions below are designed to help us know more about your lifestyle, as some lifestyle factors such as alcohol consumption and body mass index (BIM) are known to be associated with a woman's risk of breast cancer.

47. What is your weight? _______________

48. What is your height? _______________

49. On average how much moderate/intense exercise do you do in a week?

- ❑ 1 No exercise
- ❑ 2 Less than 30 minutes of exercise a week
- ❑ 3 Around 30 minutes of exercise a week
- ❑ 4 More than 30 minutes of exercise a week

50. On average how much alcohol do you drink in a day

- ❑ 1 Do not drink alcohol
- ❑ 2 Less than two standard drinks a day
- ❑ 4 Two standard drinks per day
- ❑ 5 More than two standard drinks day

Section 7: Genomic Testing Results

We would now like to know whether you have made a decision about receiving the results of your genomic testing. If you have decided to receive your test genomic testing result, the research assist for this study will contact you to arrange an appointment at the family cancer clinic.

51. Have you made a decision about receiving the results of your genomic testing for common risk variants associated with breast cancer risk?

- ☐ 1 I have decided **NOT TO RECEIVE** the results of my genomic testing. Do not contact me to arrange an appointment at a family cancer clinic.
- ☐ 2 I have decided **TO RECEIVE** the results of my genomic testing. Please contact me to arrange an appointment at a family cancer clinic.
- ☐ 3 I have **NOT DECIDED YET** as I would like **MORE INFORMATION** before making this decision. Please contact me to discuss this further.
- ☐ 4 I have **NOT DECIDED YET** and have **NO FURTHER QUESTIONS**. I will contact the research assistant for this study on 03 8559 4992 if I decide to receive my test result or have any questions in the future.

Further comments (optional):

____

____

____

____

____

____

____

____

____

YOU'RE FINISHED!

Please return the questionnaire in the enclosed reply paid envelope and post it within the next ten days, if possible

Questionnaire Two

We are interested in finding out about your recent experience receiving your genomic testing results for common risk variants associated with breast cancer risk. To help us with this, we ask that you complete the following questionnaire.

About the questionnaire

All the information you provide will be treated as strictly confidential and your identity will never be revealed in any reports. The completed questionnaires will be kept separately from any information that could identify you and will be kept securely under lock and key. There is no need for you to write your name on this questionnaire. There are no right or wrong answers, and we ask you simply to tick those answers that most apply to you.

Participation in this study is entirely voluntary; you are not obliged to participate and if you do participate you can withdraw at any time. Whatever your decision, it will not affect your relationship with your treating doctors or hospital in case you are currently being treated for breast cancer or your relationship with the researchers involved in the 'Common Genetic Variants and Familial Breast Cancer Study'.

You may feel that some of the questions we ask are stressful or upsetting. If you become upset or you do not wish to answer a question, you may skip it and go to the next question, or you may stop immediately. If you become upset or distressed as a result of your participation in this study, the research team will arrange for counselling or other appropriate support. Any counselling or support will be provided by qualified staff members who are not members of the research team. This counselling will be provided free of charge

When you have completed the questionnaire, please post the questionnaire and consent form to us, using the enclosed reply paid envelope within the next ten days, if possible.

Have you received the results of your genomic testing for breast cancer risk? (Please tick the appropriate box).

- ❑ **YES**
- ❑ **NO** ⟶ **If you answered NO, please stop here and call our Toll-free number, 1800 814 403 to speak with our Study Co-ordinator.**

Thank you very much for your help in this important study.

Registration Number: ________________

Date Issued: ________________

Section 1: Genomic Testing Result

To begin, we would like to ask about your personal experiences regarding your recent appointment at the family cancer clinic (that is, genetic counselling & genomic testing result). The answers you give are completely confidential.

1. What was the result of your genomic testing for common risk variants associated with breast cancer risk? Please note, you may choose not to disclose this information, which is fine.

- ☐ 1 I have a 'low risk score'
- ☐ 2 I have a 'high risk score'
- ☐ 3 I did not receive a definitive result
- ☐ 4 I am unsure of my genomic test result
- ☐ 5 I would prefer not to disclose my genomic testing results
- ☐ 6 Other, please specify: ________________________________

If you have a personal history of breast cancer, please answer Question 2. Otherwise, please answer Question 3.

2. In line with your personal history, did you anticipate getting this test result?

- ☐ 1 Yes, my result fits with my personal history of breast cancer
- ☐ 2 No, my result does not fit with my personal history of breast cancer
- ☐ 3 Unsure, I was not sure what to expect based on my personal history of breast cancer
- ☐ 4 I don't believe there is a relationship between my personal history and this test result
- ☐ 5 Don't know

3. According to your family history, did you anticipate getting this test result?

- ☐ 1 Yes, my result fits with my family history of breast cancer
- ☐ 2 No, my result does not fit with my family history of breast cancer
- ☐ 3 Unsure, I was not sure what to expect based on my family history of breast cancer
- ☐ 4 I don't believe there is a relationship between my family history and this test result
- ☐ 5 Don't know

4. The questions below are about some specific responses you may have experienced after receiving your genomic testing result to estimate your breast cancer risk. Please answer every question, regardless of whether you received a high or low risk.

   Please indicate whether you have experienced each statement never, rarely, sometimes, or often in the **PAST WEEK**, by circling the corresponding number.

	**Never**	**Rarely**	**Sometimes**	**Often**
Feeling upset about my test result	❑0	❑1	❑3	❑5
Feeling sad about my test result	❑0	❑1	❑3	❑5
Feeling anxious or nervous about my test result	❑0	❑1	❑3	❑5
Feeling guilty about my test result	❑0	❑1	❑3	❑5
Feeling relieved about my test result	❑0	❑1	❑3	❑5
Feeling happy about my test result	❑0	❑1	❑3	❑5
Feeling a loss of control	❑0	❑1	❑3	❑5
Having problems enjoying life because of my test result	❑0	❑1	❑3	❑5
Worrying about my risk of getting cancer [or getting cancer again]	❑0	❑1	❑3	❑5
Being uncertain about what my test result means about my cancer risk	❑0	❑1	❑3	❑5
Being uncertain about what my test result means for my child(ren) and/or family's cancer risk	❑0	❑1	❑3	❑5
Having difficulty making decisions about cancer screening or prevention (e.g., having preventive surgery or getting medical tests done)	❑0	❑1	❑3	❑5
Feeling frustrated that there are no definite cancer prevention guidelines for me	❑0	❑1	❑3	❑5
Thinking about my test results has affected my work or family life	❑0	❑1	❑3	❑5
Feeling concerned about how my test results will affect my insurance status	❑0	❑1	❑3	❑5
Having difficulty talking about my test results with family members	❑0	❑1	❑3	❑5
Feeling that my family has been supportive during the genetic counselling and testing process	❑0	❑1	❑3	❑5
Feeling satisfied with family communication about my genomic testing result	❑0	❑1	❑3	❑5
Worrying that the genetic testing process has brought about conflict within my family	❑0	❑1	❑3	❑5

Please continue over the page

Section 2: Chances of Developing Breast Cancer

In this section, we are interested to know **<u>your opinion</u>** about your chances of developing breast cancer in the future.

5. What do you think your chances of developing breast cancer sometime in the future compared to:

   a. an average person the same age & gender as you?

Much lower	Lower	Same	Higher	Much Higher
1	2	3	4	5

   b. an average person the same age & gender as you **AND** with a similar family history breast cancer as you?

Much lower	Lower	Same	Higher	Much Higher
1	2	3	4	5

6. Based on your own opinion, please rate your chances of developing breast cancer sometime in the future on a scale of 0–100%, where 0% = no chance of ever developing breast cancer, and 100% = will definitely develop breast cancer someday.

   Please do this by drawing a **<u>mark, like this:</u>** | **<u>anywhere</u>** along the line below:

0%
No chance of developing breast cancer

50%
50-50
(or 1 in 2) chance of developing breast cancer

100%
Will definitely develop breast cancer

Section 3: Knowledge of Breast Cancer Risk

In this section we would like to find out what you know about genetic variations and breast cancer risk.

7. For each of the following statements please indicate if you believe it to be True, False or Don't Know.

	False	True	Don't Know
All women at high risk for breast cancer will develop breast cancer	❑1	❑2	❑3
The interpretation of a high or low risk is the same for everyone	❑1	❑2	❑3
If a woman has a high risk for breast there are screening and preventative options available	❑1	❑2	❑3
Common risk variants associated with breast cancer risk also increases a woman's risk for ovarian cancer	❑1	❑2	❑3
There is more than one DNA change that can increase a woman's risk for breast cancer	❑1	❑2	❑3
It is possible to be diagnosed with breast cancer solely due to chance	❑1	❑2	❑3
Most women who develop breast cancer do not have family history of the disease	❑1	❑2	❑3
A woman can only inherit DNA changes associated with breast cancer risk from her mother	❑1	❑2	❑3
A woman may be at increased risk for breast cancer if she has several close relatives with breast cancer	❑1	❑2	❑3
If a woman learns that she does not have a fault in the *BRCA1* or *BRCA2* genes, that means the breast cancer in her family cannot be hereditary	❑1	❑2	❑3

Section 4: Thoughts and Feeling

8. Below is a list of comments made by people during various life events.

   Please tick the box corresponding to the statement that indicates how frequently each comment was true for you **in the past week regarding your breast cancer risk.** If any of these responses did not occur, tick the "not at all" box.

	Not at all	Rarely	Sometimes	Often
I thought about it when I didn't mean to	❑0	❑1	❑3	❑5
I avoided letting myself get upset when I thought about it or was reminded of it	❑0	❑1	❑3	❑5
I tried to remove it from my memory	❑0	❑1	❑3	❑5
I had trouble falling asleep or staying asleep because of pictures or thoughts about it that came into my mind	❑0	❑1	❑3	❑5
I had waves of strong feelings about it	❑0	❑1	❑3	❑5
I had dreams about it	❑0	❑1	❑3	❑5
I stayed away from reminders of it	❑0	❑1	❑3	❑5
I felt as if it hadn't happened or wasn't real	❑0	❑1	❑3	❑5
I tried not to talk about it	❑0	❑1	❑3	❑5
Pictures about it popped into my mind	❑0	❑1	❑3	❑5
Other things kept making me think about it	❑0	❑1	❑3	❑5
I was aware that I still had a lot of feelings about it, but I didn't deal with them	❑0	❑1	❑3	❑5
I tried not to think about it	❑0	❑1	❑3	❑5
Any reminder brought back feelings about it	❑0	❑1	❑3	❑5
My feelings about it were kind of numb	❑0	❑1	❑3	❑5

Please continue over the page

This section is designed to help us know how you feel. Please read each item below, and place a tick in the box opposite the reply, which comes closest to how you have been feeling in the **PAST WEEK**. Don't take too long over your replies; your immediate reaction to each item will probably be more accurate than a long thought out response.

9. I feel tense or 'wound up':
   - ❑ 1 Most of the time
   - ❑ 2 A lot of the time
   - ❑ 3 From time to time, occasionally
   - ❑ 4 Not at all

10. I still enjoy the things I used to enjoy:
   - ❑ 1 Definitely as much
   - ❑ 2 Not quite so much
   - ❑ 3 Only a little
   - ❑ 4 Hardly at all

11. I get a sort of frightened feeling as if something awful is about to happen:
   - ❑ 1 Very definitively and quite badly
   - ❑ 2 Yes, but not too badly
   - ❑ 3 A little, but it doesn't worry me
   - ❑ 4 Not at all

12. I can laugh and see the funny side of things:
   - ❑ 1 As much as I always could
   - ❑ 2 Not quite so much
   - ❑ 3 Definitely not so much now
   - ❑ 4 Not at all

13. Worrying thoughts go through my mind:
   - ❑ 1 A great deal of the time
   - ❑ 2 A lot of the time
   - ❑ 3 From time to time but not too often
   - ❑ 4 Only occasionally

14. I feel cheerful:
   - ❑ 1 Not at all
   - ❑ 2 Not often
   - ❑ 3 Sometimes
   - ❑ 4 Most of the time

15. I can sit at ease and feel relaxed:
   - ❑ 1 Definitely
   - ❑ 2 Usually
   - ❑ 3 Not often
   - ❑ 4 Not at all

16. I feel as if I am slowed down:
   - ❑ 1 Nearly all the time
   - ❑ 2 Very often
   - ❑ 3 Sometimes
   - ❑ 4 Not at all

17. I get a sort of frightened feeling like butterflies in my stomach:
   - ❑ 1 Not at all
   - ❑ 2 Occasionally
   - ❑ 3 Quite often
   - ❑ 4 Very often

18. I have lost interest in my appearance:
   - ❑ 1 Definitely
   - ❑ 2 I don't take so much care as I should
   - ❑ 3 I may not take quite as much care
   - ❑ 4 I take just as much care as ever

19. I feel restless as if I have to be on the move:
   - ❑ 1 Very much indeed
   - ❑ 2 Quite a lot
   - ❑ 3 Not very much
   - ❑ 4 Not at all

20. I look forward with enjoyment to things:
   - ❑ 1 As much as I ever did
   - ❑ 2 Rather less than I used to
   - ❑ 3 Definitely less than I used to
   - ❑ 4 Hardly at all

21. I get sudden feelings of panic:
   - ❑ 1 Very often indeed
   - ❑ 2 Quite often
   - ❑ 3 Not very often
   - ❑ 4 Not at all

22. I can enjoy a good book or radio or TV program:
   - ❑ 1 Often
   - ❑ 2 Sometimes
   - ❑ 3 Not often
   - ❑ 4 Very seldom

Section 5: Breast Cancer Risk Management

In this section we would like to know how you plan to manage your breast cancer risk since receiving your genomic testing results. Not all options may be relevant to you. However, they may have been discussed with you during an appointment at the family cancer clinic. Please note, that your responses to the following questions, will not limit your options in the future.

23. During your appointment at the family cancer clinic, were you advised to have screening tests for breast cancer?

- ❑ 1 Not applicable, I had a bilateral mastectomy (please go to question 26)
- ❑ 3 Unsure
- ❑ 4 Yes, I was advised to have breast screening but I don't need to start yet
- ❑ 5 Yes, I was advised start breast screening in the next 12 months

→ Please indicate below how likely you are to organise the recommended screening in the next 12 months:

Not at all likely	Somewhat likely	Unsure	Quite likely	Extremely likely
1	2	3	4	5

24. During your appointment at the family cancer clinic, were you told about the option to have a bilateral mastectomy (surgery to remove both breast) to reduce your breast cancer risk?

- ❑ 1 No, I was not told about this option
- ❑ 2 Unsure
- ❑ 4 Yes, I was told about this option

→ Please indication below how likely you are to have this surgery in the next 12 months:

Not at all likely	Somewhat likely	Unsure	Quite likely	Extremely likely
1	2	3	4	5

25. During your appointment at the family cancer clinic, were you advised about the option to take medication (e.g. Tamoxifen) to reduce your breast cancer risk?

- ❑ 1 No, I was not told about this option
- ❑ 2 Unsure
- ❑ 4 Yes, I was told about this option

→ Please indication below how likely you are to take medication risk reducing in the next 12 months:

Not at all likely	Somewhat likely	Unsure	Quite likely	Extremely likely
1	2	3	4	5

Section 6: Decision to Receive Test Result

26. Please reflect on you decision to receive your genomic testing result for common risk variants associated with breast cancer risk.

    Please respond to each of the following statements in the table below by ticking the number that best reflects how you feel.

	Strongly disagree	Disagree	Neither disagree or agree	Agree	Strongly agree
It was the right decision	❑1	❑2	❑3	❑4	❑5
I regret the choice that was made	❑1	❑2	❑3	❑4	❑5
I would make the same choice if I had to do it over again	❑1	❑2	❑3	❑4	❑5
The choice did me a lot of harm	❑1	❑2	❑3	❑4	❑5
The decision was a wise one	❑1	❑2	❑3	❑4	❑5

Further comments (optional):

____

____

____

____

____

____

____

____

You're finished!

Please return the questionnaire in the enclosed reply paid envelope and post it within the next ten days, if possible.

Questionnaire Three – Receivers

We are interested in understanding your experience in the past 12 months since receiving your genomic testing results for common risk variants associated with breast cancer. To help us with this, we ask that you complete the following questionnaire.

About the questionnaire

All the information you provide will be treated as strictly confidential and your identity will never be revealed in any reports. The completed questionnaires will be kept separately from any information that could identify you and will be kept securely under lock and key. There is no need for you to write your name on this questionnaire. There are no right or wrong answers, and we ask you simply to tick those answers that most apply to you.

Participation in this study is entirely voluntary; you are not obliged to participate and if you do participate you can withdraw at any time. Whatever your decision, it will not affect your relationship with your treating doctors or hospital in case you are currently being treated for breast cancer. It will also not affect your relationship with the researchers involved in the 'Common Genetic Variants and Familial Breast Cancer Study'.

You may feel that some of the questions we ask are stressful or upsetting. If you become upset or you do not wish to answer a question, you may skip it and go to the next question, or you may stop immediately. If you become upset or distressed as a result of your participation in this study, the research team will arrange for counselling or other appropriate support. Any counselling or support will be provided by qualified staff members who are not members of the research team. This counselling will be provided free of charge

When you have completed the questionnaire, please post the questionnaire using the enclosed reply paid envelope within the next ten days, if possible.

Have you received the results of your genomic testing for breast cancer risk? (Please tick the appropriate box).

- ❑ **YES**
- ❑ **NO** ⟶ **If you answered NO, please stop here and call our Toll-free number, 1800 814 403 to speak with our Study Co-ordinator.**

Thank you very much for your help in this important study.

Registration Number: ________________

Date Issued: ________________

Section 1: Genomic Testing Result

To begin, we would like to ask about your personal experiences regarding your recent appointment at the family cancer clinic (that is, genetic counselling & genomic testing result). The answers you give are completely confidential.

1. What was the result of your genomic testing for common risk variants associated with breast cancer risk? Please note, you may choose not to disclose this information, which is fine.

   - ☐ 1 I have a 'low risk score'
   - ☐ 2 I have a 'high risk score'
   - ☐ 3 I did not receive a definitive result
   - ☐ 4 I am unsure of my genomic test result
   - ☐ 5 I would prefer not to disclose my genomic testing results
   - ☐ 6 Other, please specify: ___________________________________

Section 2: Chances of Developing Breast Cancer

In this section, we are interested to know **your opinion** about your chances of developing breast cancer in the future.

2. What do you think are your chances of developing breast cancer sometime in the future compared to:

   a. an average person the same age and gender as you?

Much lower	Lower	Same	Higher	Much Higher
1	2	3	4	5

   b. an average person the same age and gender as you **AND** with a similar family history breast cancer as you?

Much lower	Lower	Same	Higher	Much Higher
1	2	3	4	5

3. Based on your own opinion, please rate your chances of developing breast cancer sometime in the future on a scale of 0–100%, where 0% = no chance of ever developing breast cancer, and 100% = will definitely develop breast cancer someday.

   Please do this by drawing a **mark, like this:** | **anywhere** along the line below:

0%	50%	100%
No chance of developing breast cancer	50-50 (or 1 in 2) chance of developing breast cancer	Will definitely develop breast cancer

Section 3: Knowledge of Breast Cancer Risk

In this section we would like to find out what you know about genetic variations and breast cancer risk.

4. For each of the following statements please indicate if you believe it to be True, False or Don't Know. If you are unsure or don't know the answer, please tick the "don't know" box.

	False	True	Don't Know
All women at high risk for breast cancer will develop breast cancer	❑1	❑2	❑3
The interpretation of a high or low risk is the same for everyone	❑1	❑2	❑3
If a woman has a high risk for breast there are screening and preventative options available	❑1	❑2	❑3
Common risk variants associated with breast cancer risk also increases a woman's risk for ovarian cancer	❑1	❑2	❑3
There is more than one DNA change that can increase a woman's risk for breast cancer	❑1	❑2	❑3
It is possible to be diagnosed with breast cancer solely due to chance	❑1	❑2	❑3
Most women who develop breast cancer do not have family history of the disease	❑1	❑2	❑3
A woman can only inherit DNA changes associated with breast cancer risk from her mother	❑1	❑2	❑3
A woman may be at increased risk for breast cancer if she has several close relatives with breast cancer	❑1	❑2	❑3
If a woman learns that she does not have a fault in the *BRCA1* or *BRCA2* genes, that means the breast cancer in her family cannot be hereditary	❑1	❑2	❑3

Section 4: Thoughts and Feelings

5. Below is a list of comments made by people during various life events.

   Please tick the box corresponding to the statement that indicates how frequently each comment was true for you in the **past week regarding your risk of developing breast cancer**. If any of these responses did not occur, tick the "not at all" box.

	**Not at all**	**Rarely**	**Sometimes**	**Often**
I thought about it when I didn't mean to	❑0	❑1	❑3	❑5
I avoided letting myself get upset when I thought about it or was reminded of it	❑0	❑1	❑3	❑5
I tried to remove it from my memory	❑0	❑1	❑3	❑5
I had trouble falling asleep or staying asleep because of pictures or thoughts about it that came into my mind	❑0	❑1	❑3	❑5
I had waves of strong feelings about it	❑0	❑1	❑3	❑5
I had dreams about it	❑0	❑1	❑3	❑5
I stayed away from reminders of it	❑0	❑1	❑3	❑5
I felt as if it hadn't happened or wasn't real	❑0	❑1	❑3	❑5
I tried not to talk about it	❑0	❑1	❑3	❑5
Pictures about it popped into my mind	❑0	❑1	❑3	❑5
Other things kept making me think about it	❑0	❑1	❑3	❑5
I was aware that I still had a lot of feelings about it, but I didn't deal with them	❑0	❑1	❑3	❑5
I tried not to think about it	❑0	❑1	❑3	❑5
Any reminder brought back feelings about it	❑0	❑1	❑3	❑5
My feelings about it were kind of numb	❑0	❑1	❑3	❑5

Office use only:
Score I:_______
Score A:_______
Total score:____

6. The questions below are about some specific responses you may have had after receiving your genomic test results.

   Please indicate whether you have experienced each statement never, rarely, sometimes, or often in the **PAST WEEK**, by circling the corresponding number.

	**Never**	**Rarely**	**Sometimes**	**Often**
Feeling upset about my test result	❑0	❑1	❑3	❑5
Feeling sad about my test result	❑0	❑1	❑3	❑5
Feeling anxious or nervous about my test result	❑0	❑1	❑3	❑5
Feeling guilty about my test result	❑0	❑1	❑3	❑5
Feeling relieved about my test result	❑0	❑1	❑3	❑5
Feeling happy about my test result	❑0	❑1	❑3	❑5
Feeling a loss of control	❑0	❑1	❑3	❑5
Having problems enjoying life because of my test result	❑0	❑1	❑3	❑5
Worrying about my risk of getting cancer [or getting cancer again]	❑0	❑1	❑3	❑5
Being uncertain about what my test result means about my cancer risk	❑0	❑1	❑3	❑5
Being uncertain about what my test result means for my child(ren) and/or family's cancer risk	❑0	❑1	❑3	❑5
Having difficulty making decisions about cancer screening or prevention (e.g., having preventive surgery or getting medical tests done)	❑0	❑1	❑3	❑5
Feeling frustrated that there are no definite cancer prevention guidelines for me	❑0	❑1	❑3	❑5
Thinking about my test results has affected my work or family life	❑0	❑1	❑3	❑5
Feeling concerned about how my test results will affect my insurance status	❑0	❑1	❑3	❑5
Having difficulty talking about my test results with family members	❑0	❑1	❑3	❑5
Feeling that my family has been supportive during the genetic counselling and testing process	❑0	❑1	❑3	❑5
Feeling satisfied with family communication about my genomic testing result	❑0	❑1	❑3	❑5
Worrying that the genetic testing process has brought about conflict within my family	❑0	❑1	❑3	❑5

Office use only:
Score D:______
Score P: ______
Total score:___

This section is designed to help us know how you feel. Please read each item below, and place a tick in the box opposite the reply, which comes closest to how you have been feeling in the **PAST WEEK**. Don't take too long over your replies; your immediate reaction to each item will probably be more accurate than a long thought out response.

7. I feel tense or 'wound up':
   - ❑ 1 Most of the time
   - ❑ 2 A lot of the time
   - ❑ 3 From time to time, occasionally
   - ❑ 4 Not at all

8. I still enjoy the things I used to enjoy:
   - ❑ 1 Definitely as much
   - ❑ 2 Not quite so much
   - ❑ 3 Only a little
   - ❑ 4 Hardly at all

9. I get a sort of frightened feeling as if something awful is about to happen:
   - ❑ 1 Very definitively and quite badly
   - ❑ 2 Yes, but not too badly
   - ❑ 3 A little, but it doesn't worry me
   - ❑ 4 Not at all

10. I can laugh and see the funny side of things:
    - ❑ 1 As much as I always could
    - ❑ 2 Not quite so much
    - ❑ 3 Definitely not so much now
    - ❑ 4 Not at all

11. Worrying thoughts go through my mind:
    - ❑ 1 A great deal of the time
    - ❑ 2 A lot of the time
    - ❑ 3 From time to time but not too often
    - ❑ 4 Only occasionally

12. I feel cheerful:
    - ❑ 1 Not at all
    - ❑ 2 Not often
    - ❑ 3 Sometimes
    - ❑ 4 Most of the time

13. I can sit at ease and feel relaxed:
    - ❑ 1 Definitely
    - ❑ 2 Usually
    - ❑ 3 Not often
    - ❑ 4 Not at all

14. I feel as if I am slowed down:
    - ❑ 1 Nearly all the time
    - ❑ 2 Very often
    - ❑ 3 Sometimes
    - ❑ 4 Not at all

15. I get a sort of frightened feeling like butterflies in my stomach:
    - ❑ 1 Not at all
    - ❑ 2 Occasionally
    - ❑ 3 Quite often
    - ❑ 4 Very often

16. I have lost interest in my appearance:
    - ❑ 1 Definitely
    - ❑ 2 I don't take so much care as I should
    - ❑ 3 I may not take quite as much care
    - ❑ 4 I take just as much care as ever

17. I feel restless as if I have to be on the move:
    - ❑ 1 Very much indeed
    - ❑ 2 Quite a lot
    - ❑ 3 Not very much
    - ❑ 4 Not at all

18. I look forward with enjoyment to things:
    - ❑ 1 As much as I ever did
    - ❑ 2 Rather less than I used to
    - ❑ 3 Definitely less than I used to
    - ❑ 4 Hardly at all

19. I get sudden feelings of panic:
    - ❑ 1 Very often indeed
    - ❑ 2 Quite often
    - ❑ 3 Not very often
    - ❑ 4 Not at all

20. I can enjoy a good book or radio or TV program:
    - ❑ 1 Often
    - ❑ 2 Sometimes
    - ❑ 3 Not often
    - ❑ 4 Very seldom

Office use only:
Score A:______
Score D:______

21. Below is a list various stress life events that that people can experience.

Please tick the box below, either 'Yes' or 'No' to indicate if any of the events below occurred for **you in the past year.**

	No	Yes
You yourself suffered a serious illness (including a new cancer diagnosis), injury or an assault	❑1	❑2
A serious illness (including cancer diagnosis), injury or assault happened to a close relative	❑1	❑2
Your parent, child or spouse died	❑1	❑2
A close family friend or another relative (aunt, cousin, grandparent) died	❑1	❑2
You had a separation due to marital difficulties	❑1	❑2
You broke off a steady relationship	❑1	❑2
You had a serious problem with a close friend, neighbour or relative	❑1	❑2
You became unemployed or you were seeking work unsuccessfully for more than one month	❑1	❑2
You were sacked from your job	❑1	❑2
You had a major financial crisis	❑1	❑2
You had problems with the police and a court appearance	❑1	❑2
You moved to a new house	❑1	❑2
Something you valued was lost or stolen	❑1	❑2

Section 5: Breast Cancer Screening and Preventative Strategies

This section looks at your current strategies to manage your breast cancer risk. Not all options may be relevant to you. However, they have been discussed with you during an appointment at the family cancer clinic. Please note, that your responses to the following questions, will not limit your options in the future.

22. Have you had a bilateral mastectomy (surgery to remove both breasts)?

- ☐ 1 No
- ☐ 2 Yes

23. Do you currently take medication to reduce your breast cancer risk (e.g. Tamoxifen)?

- ☐ 1 No
- ☐ 2 Yes

24. Do you examine your breasts yourself?

- ☐ 1 No, please go to question 26
- ☐ 2 Yes

25. If you have answered yes, how often have you examined your breasts over the **past year**?

- ☐ 1 Less frequently than monthly
- ☐ 2 Monthly
- ☐ 3 More frequently than monthly

26. Have you ever had a doctor or nurse examine your breasts over the **past year**?

- ☐ 1 No, please go to question 28
- ☐ 2 Yes

27. If you have answered yes, how often have you had a doctor or nurse examine your breasts over the **past year**?

- ☐ 1 Less frequently than once a year
- ☐ 2 Once a year
- ☐ 3 More frequently than once year

28. Have you ever had any screening tests for breast cancer? Please tick any that apply.

- ☐ 1 Not applicable, (had double mastectomy), please go question 30
- ☐ 2 No, please go question 30
- ☐ 3 Yes, I have had screening tests for breast cancer

29. If you have answered yes, please indicate what screening test(s) you have and the year of your most recent test:

- ☐ 1 Mammogram (an X-ray of the breast for women with no breast symptoms)
  Year: ________________
- ☐ 2 Breast ultrasound
  Year: ________________
- ☐ 3 Breast MRI (or magnetic resonance imaging, a test that uses a magnetic field to view the breast tissue).
  Year: ________________

The questions below are designed to help us know more about your lifestyle, as some lifestyle factors such as alcohol consumption and body mass index (BIM) are known to be associated with a woman's risk of breast cancer.

30. What is your weight? _______________

31. What if your height? ____________

32. On average how much moderate/intense exercise do you do in a week?

- ❑ 1 No exercise
- ❑ 2 Less than 30 minutes of exercise a week
- ❑ 3 Around 30 minutes of exercise a week
- ❑ 4 More than 30 minutes of exercise a week

33. On average how much alcohol do you drink in a day?

- ❑ 1 Do not drink alcohol
- ❑ 2 Less than two standard drinks a day
- ❑ 4 Two stand drinks per day
- ❑ 5 More than two standard drinks day

Section 6: Decision about Receiving Test Result

34. Please reflect on your decision to receive your genomic testing result for common risk variants associated with breast cancer risk.

    Please respond to each of the following statements in the table below by selecting the number that best reflects how you feel.

	**Strongly disagree**	**Disagree**	**Neither disagree or agree**	**Agree**	**Strongly agree**
It was the right decision	❑1	❑2	❑3	❑4	❑5
I regret the choice that was made	❑1	❑2	❑3	❑4	❑5
I would make the same choice if I had to do it over again	❑1	❑2	❑3	❑4	❑5
The choice did me a lot of harm	❑1	❑2	❑3	❑4	❑5
The decision was a wise one	❑1	❑2	❑3	❑4	❑5

Further comments (optional):

____

____

____

____

____

____

____

____

____

You’re finished!

Please return the questionnaire in the enclosed reply paid envelope and post it within the next ten days, if possible.

Questionnaire Three - Decliners

We are interested in understanding the experiences of people who have chosen **not** to receive their genomic testing result. To help us with this, we ask that you complete the following questionnaire.

About the questionnaire

All the information you provide will be treated as strictly confidential and your identity will never be revealed in any reports. The completed questionnaires will be kept separately from any information that could identify you and will be kept securely under lock and key. There is no need for you to write your name on this questionnaire. There are no right or wrong answers, and we ask you simply to tick those answers that most apply to you.

Participation in this study is entirely voluntary; you are not obliged to participate and if you do participate you can withdraw at any time. Whatever your decision, it will not affect your relationship with your treating doctors or hospital in case you are currently being treated for breast cancer. It will also not affect your relationship with the researchers involved in the 'Common Genetic Variants and Familial Breast Cancer Study'.

You may feel that some of the questions we ask are stressful or upsetting. If you become upset or you do not wish to answer a question, you may skip it and go to the next question, or you may stop immediately. If you become upset or distressed as a result of your participation in this study, the research team will arrange for counselling or other appropriate support. Any counselling or support will be provided by qualified staff members who are not members of the research team. This counselling will be provided free of charge

When you have completed the questionnaire, please post the questionnaire and consent form to us, using the enclosed reply paid envelope within the next ten days, if possible.

Have you received the results of your genomic testing for breast cancer risk? (Please tick the appropriate box).

- ❑ **NO**
- ❑ **YES** ⟶ **If you answered NO, please stop here and call our Toll-free number, 1800 814 403 to speak with our Study Co-ordinator.**

Thank you very much for your help in this important study.

Registration Number: _______________

Date Issued: _______________

Section 1: Thoughts and Feeling

1. Below is a list of comments made by people during various life events.

   Please tick the box corresponding to the statement that indicates how frequently each comment was true for you **in the past week regarding your risk of developing breast cancer.** If any of these responses did not occur, tick the "not at all" box.

	Not at all	Rarely	Sometimes	Often
I thought about it when I didn't mean to	❑0	❑1	❑3	❑5
I avoided letting myself get upset when I thought about it or was reminded of it	❑0	❑1	❑3	❑5
I tried to remove it from my memory	❑0	❑1	❑3	❑5
I had trouble falling asleep or staying asleep because of pictures or thoughts about it that came into my mind	❑0	❑1	❑3	❑5
I had waves of strong feelings about it	❑0	❑1	❑3	❑5
I had dreams about it	❑0	❑1	❑3	❑5
I stayed away from reminders of it	❑0	❑1	❑3	❑5
I felt as if it hadn't happened or wasn't real	❑0	❑1	❑3	❑5
I tried not to talk about it	❑0	❑1	❑3	❑5
Pictures about it popped into my mind	❑0	❑1	❑3	❑5
Other things kept making me think about it	❑0	❑1	❑3	❑5
I was aware that I still had a lot of feelings about it, but I didn't deal with them	❑0	❑1	❑3	❑5
I tried not to think about it	❑0	❑1	❑3	❑5
Any reminder brought back feelings about it	❑0	❑1	❑3	❑5
My feelings about it were kind of numb	❑0	❑1	❑3	❑5

Office use only:
Score I:_______
Score A:__
Total score:___

This section is designed to help us know how you feel. Please read each item below, and place a tick in the box opposite the reply, which comes closest to how you have been feeling in the **PAST WEEK**. Don't take too long over your replies; your immediate reaction to each item will probably be more accurate than a long thought out response.

2. I feel tense or 'wound up':
   - ☐ 1 Most of the time
   - ☐ 2 A lot of the time
   - ☐ 3 From time to time, occasionally
   - ☐ 4 Not at all

3. I still enjoy the things I used to enjoy:
   - ☐ 1 Definitely as much
   - ☐ 2 Not quite so much
   - ☐ 3 Only a little
   - ☐ 4 Hardly at all

4. I get a sort of frightened feeling as if something awful is about to happen:
   - ☐ 1 Very definitively and quite badly
   - ☐ 2 Yes, but not too badly
   - ☐ 3 A little, but it doesn't worry me
   - ☐ 4 Not at all

5. I can laugh and see the funny side of things:
   - ☐ 1 As much as I always could
   - ☐ 2 Not quite so much
   - ☐ 3 Definitely not so much now
   - ☐ 4 Not at all

6. Worrying thoughts go through my mind:
   - ☐ 1 A great deal of the time
   - ☐ 2 A lot of the time
   - ☐ 3 From time to time but not too often
   - ☐ 4 Only occasionally

7. I feel cheerful:
   - ☐ 1 Not at all
   - ☐ 2 Not often
   - ☐ 3 Sometimes
   - ☐ 4 Most of the time

8. I can sit at ease and feel relaxed:
   - ☐ 1 Definitely
   - ☐ 2 Usually
   - ☐ 3 Not often
   - ☐ 4 Not at all

9. I feel as if I am slowed down:
   - ☐ 1 Nearly all the time
   - ☐ 2 Very often
   - ☐ 3 Sometimes
   - ☐ 4 Not at all

10. I get a sort of frightened feeling like butterflies in my stomach:
    - ☐ 1 Not at all
    - ☐ 2 Occasionally
    - ☐ 3 Quite often
    - ☐ 4 Very often

11. I have lost interest in my appearance:
    - ☐ 1 Definitely
    - ☐ 2 I don't take so much care as I should
    - ☐ 3 I may not take quite as much care
    - ☐ 4 I take just as much care as ever

12. I feel restless as if I have to be on the move:
    - ☐ 1 Very much indeed
    - ☐ 2 Quite a lot
    - ☐ 3 Not very much
    - ☐ 4 Not at all

13. I look forward with enjoyment to things:
    - ☐ 1 As much as I ever did
    - ☐ 2 Rather less than I used to
    - ☐ 3 Definitely less than I used to
    - ☐ 4 Hardly at all

14. I get sudden feelings of panic:
    - ☐ 1 Very often indeed
    - ☐ 2 Quite often
    - ☐ 3 Not very often
    - ☐ 4 Not at all

15. I can enjoy a good book or radio or TV program:
    - ☐ 1 Often
    - ☐ 2 Sometimes
    - ☐ 3 Not often
    - ☐ 4 Very seldom

Office use only:
Score A:______
Score D:______

16. Below is a list various stress life events that that people can experience.

Please tick the box below, either 'Yes' or 'No' to indicate if any of the events below occurred for **you in the past year.**

	No	Yes
You yourself suffered a serious illness (including a new cancer diagnosis), injury or an assault	❑0	❑1
A serious illness (including cancer diagnosis), injury or assault happened to a close relative	❑0	❑1
Your parent, child or spouse died	❑0	❑1
A close family friend or another relative (aunt, cousin, grandparent) died	❑0	❑1
You had a separation due to marital difficulties	❑0	❑1
You broke off a steady relationship	❑0	❑1
You had a serious problem with a close friend, neighbour or relative	❑0	❑1
You became unemployed or you were seeking work unsuccessfully for more than one month	❑0	❑1
You were sacked from your job	❑0	❑1
You had a major financial crisis	❑0	❑1
You had problems with the police and a court appearance	❑0	❑1
You moved to a new house	❑0	❑1
Something you valued was lost or stolen	❑0	❑1

Section 2: Screening for breast cancer and Preventative Strategies

This section looks at your current strategies to manage your breast cancer risk. Not all options may be relevant to you. Please note, that your responses to the following questions, will not limit your options in the future.

17. Have you had a bilateral mastectomy (surgery to remove both breasts)?

- ❑ 1 No
- ❑ 2 Yes

18. Do you currently take medication to reduce your breast cancer risk (e.g. Tamoxifen)?

- ❑ 1 No
- ❑ 2 Yes

19. Do you examine your breasts yourself?

- ❑ 1 No, please go to question 21
- ❑ 2 Yes

20. If you have answered yes, how often have you examined your breasts over the **past year**?

- ❑ 1 Less frequently than monthly
- ❑ 2 Monthly
- ❑ 3 More frequently than monthly

21. Have you ever had a doctor or nurse examine your breasts?

- ❑ 1 No, please go to question 23
- ❑ 2 Yes

22. If you have answered yes, how often have you had a doctor or nurse examine your breasts over the **past year**?

- ❑ 1 Less frequently than once a year
- ❑ 2 Once a year
- ❑ 3 More frequently than once year

23. Have you ever had any screening tests for breast cancer? Please tick any that apply.

- ❑ 1 Not applicable, (had double mastectomy), please go question 25
- ❑ 2 No, please go question 25
- ❑ 3 Yes, I have had screening tests for breast cancer

24. If you have answered yes, please indicate what screening test(s) you have and the year of your most recent test:

- ❑ 1 Mammogram (an X-ray of the breast for women with no breast symptoms)
  Year: ________________
- ❑ 2 Breast ultrasound
  Year: ________________
- ❑ 3 Breast MRI (or magnetic resonance imaging, a test that uses a magnetic field to view the breast tissue).
  Year: ________________

The questions below are designed to help us know more about your lifestyle, as some lifestyle factors such as alcohol consumption and body mass index (BIM) are known to be associated with a woman's risk of breast cancer.

25. What is your weight? _______________

26. What if your height? ____________

27. On average how much moderate/intense exercise do you do in a week?

- ☐ 1 No exercise
- ☐ 2 Less than 30 minutes of exercise a week
- ☐ 3 Around 30 minutes of exercise a week
- ☐ 4 More than 30 minutes of exercise a week

28. On average how much alcohol do you drink in a day?

- ☐ 1 Do not drink alcohol
- ☐ 2 Less than two standard drinks a day
- ☐ 4 Two stand drinks per day
- ☐ 5 More than two standard drinks day

Section 3: Genomic testing

29. The table below lists common reasons for why people may choose not to receive the results of their genomic testing. Please rate the degree to which each of these reasons applies to you by circling the number that best represents your experience.

    I have not received my genomic testing results because…

	Not Applicable	Disagree	Neither agree or disagree	Agree
I am happy with life right now	❑0	❑1	❑4	❑3
I am too young to know my test result	❑0	❑1	❑4	❑3
The test will not tell me when I will develop breast cancer	❑0	❑1	❑4	❑3
I feel I already know my chances of developing breast cancer	❑0	❑1	❑4	❑3
I have screening regularly and so I don’t need the result	❑0	❑1	❑4	❑3
I am worried about the influence of the test result on my relationship with my partner	❑0	❑1	❑4	❑3
It might impact my finances including mortgage, employability and/or insurance	❑0	❑1	❑4	❑3
I have not had time	❑0	❑1	❑4	❑3
The nearest Familial Cancer Clinic is too far away for me travel	❑0	❑1	❑4	❑3
There is no treatment for breast cancer	❑0	❑1	❑4	❑3
I am worried about how I would discuss my result with my family	❑0	❑1	❑4	❑3
I am too old	❑0	❑1	❑4	❑3
I am concerned I may not be able to cope if I am found to have a high polygenic risk for breast cancer	❑0	❑1	❑4	❑3
I am concerned about the reactions of my children	❑0	❑1	❑4	❑3

Are there any other reasons why you may have chosen no to receive you genomic test results (please specify):

____

____

____

____

 Please continue over the page

Section 4: Decision about Not Receiving Test Result

30. Please reflect on your decision **NOT** to receive your genomic testing result for common risk variants associated with breast cancer risk.

    Please respond to each of the following statements in the table below by selecting the number that best reflects how you feel.

	**Strongly disagree**	**Disagree**	**Neither disagree or agree**	**Agree**	**Strongly agree**
It was the right decision	❑1	❑2	❑3	❑4	❑5
I regret the choice that was made	❑1	❑2	❑3	❑4	❑5
I would make the same choice if I had to do it over again	❑1	❑2	❑3	❑4	❑5
The choice did me a lot of harm	❑1	❑2	❑3	❑4	❑5
The decision was a wise one	❑1	❑2	❑3	❑4	❑5

Thank you for completing the questionnaire!

Further comments (optional):

____

____

____

____

____

____

____

____

____

You're finished!

Please return the questionnaire in the enclosed reply paid envelope and post it within the next ten days, if possible.
